# Hypoxia potentiates gemcitabine-induced stemness in pancreatic cancer cells through AKT/Notch1 signaling

**DOI:** 10.1186/s13046-018-0972-3

**Published:** 2018-11-28

**Authors:** Zhengle Zhang, Han Han, Yuping Rong, Kongfan Zhu, Zhongchao Zhu, Zhigang Tang, Chenglong Xiong, Jing Tao

**Affiliations:** 1 Department of Pancreatic Surgery, Renmin Hospital, Wuhan University, 238 Jiefang Road, Wuhan, 430060 Hubei Province China; 2 Department of Dermatology, Central Hospital of Wuhan, Tongji Medical College, Huazhong University of Science and Technology, Wuhan, 430014 Hubei Province China

**Keywords:** Gemcitabine, Hypoxia, Cancer stem cell, AKT, Notch1

## Abstract

**Background:**

Profound chemoresistance remains an intractable obstacle in pancreatic cancer treatment. Pancreatic cancer stem cells (CSCs) and the ubiquitous hypoxic niche have been proposed to account for drug resistance. However, the mechanism involved requires further exploration. This study investigated whether the hypoxic niche enhances gemcitabine-induced stemness and acquired resistance in pancreatic cancer cells by activating the AKT/Notch1 signaling cascade. The therapeutic effects of blockading this signaling cascade on gemcitabine-enriched CSCs were also investigated.

**Methods:**

The expression levels of CSC-associated markers Bmi1 and Sox2 as well as those of proteins involved in AKT/Notch1 signaling were measured by Western blot analysis. The expression level of the pancreatic CSC marker CD24 was measured by flow cytometry. Change in gemcitabine sensitivity was evaluated by the MTT assay. The ability of sphere formation was tested by the sphere-forming assay in stem cell medium. The ability of migration and invasion was detected by the transwell migration/invasion assay. A mouse xenograft model of pancreatic cancer was established to determine the effect of Notch1 inhibition on the killing effect of gemcitabine in vivo. The ability of metastasis was investigated by an in vivo lung metastasis assay.

**Results:**

Gemcitabine promoted pancreatic cancer cell stemness and associated malignant phenotypes such as enhanced migration, invasion, metastasis, and chemoresistance. The AKT/Notch1 signaling cascade was activated after gemcitabine treatment and mediated this process. Blockading this pathway enhanced the killing effect of gemcitabine in vivo. However, supplementation with hypoxia treatment synergistically enhanced the AKT/Notch1 signaling pathway and collaboratively promoted gemcitabine-induced stemness.

**Conclusions:**

These findings demonstrate a novel mechanism of acquired gemcitabine resistance in pancreatic cancer cells through induction of stemness, which was mediated by the activation of AKT/Notch1 signaling and synergistically aggravated by the ubiquitous hypoxic niche. Our results might provide new insights for identifying potential targets for reversing chemoresistance in patients with pancreatic cancer.

**Electronic supplementary material:**

The online version of this article (10.1186/s13046-018-0972-3) contains supplementary material, which is available to authorized users.

## Background

Pancreatic cancer is one of the most lethal cancers worldwide, with a 5-year survival rate that has remained at less than 10% for the past few decades [1, 2]. For many patients, there is little choice other than chemotherapy, especially in the advanced stage [3]. However, gemcitabine, a first-line anticancer drug for pancreatic cancer, provides a limited survival advantage in treated patients [4]. Numerous strategies have been proposed to improve the therapeutic effect of gemcitabine, but the prognosis for patients with pancreatic cancer remains disappointing [5]. Therefore, identifying new chemotherapeutic agents or adjuvant therapies is necessary to enhance the effectiveness of chemotherapy and reduce tumor recurrence.

Accumulating evidence indicates that tumors harbor a subpopulation of cells, termed cancer stem cells (CSCs), that are responsible for initiating tumor growth and driving relapse after chemotherapy [6, 7]. CSC-associated markers Bmi1 and Sox2 sufficiently enhance self-renewal and dedifferentiation and endow pancreatic cancer cells with stemness [8, 9]. Further, the pancreatic CSC marker CD24 increases the ability of cells to migrate and invade and has a close correlation with a poor prognosis [10–12]. Our previous results suggested that gemcitabine can enhance the stemness of pancreatic cancer cells [13]; however, the exact mechanism remains to be determined. Clarifying the mechanism involved in this process will help identify adjuvant agents for enhancing the killing effect of gemcitabine chemotherapy.

A hypoxic microenvironment has been verified in many malignances, including pancreatic cancer; it plays a critical role in the resistance of cancer cells to anticancer drugs [14, 15]. Recent studies have shown that, under hypoxic conditions, pancreatic cancer cells exhibit substantial apoptosis resistance induced by gemcitabine [14]; however, the mechanism remains elusive. Hypoxia signaling has a close correlation with the induction and maintenance of stemness phenotypes, such as self-renewal, undifferentiated condition, sphere-forming ability [16–18]. In a preliminary study, we also found that hypoxia promotes the expression of CSC-associated markers Bmi1 and Sox2 in pancreatic cancer cells. We, therefore, speculate that the hypoxic niche might synergistically enhance the acquired gemcitabine chemoresistance through stemness induction.

Notch signaling is evolutionarily conserved and critically implicated in cell fate, including proliferation, differentiation, and apoptosis [19, 20]. Mounting evidence indicates that the release of the Notch intracellular domain (NICD) from its ligands leads to aberrant activation of Notch signaling in a variety of malignancies [21–23]. Reports concerning the correlation between Notch1 signaling and CSC phenotype have increased in recent times. It has been suggested that aberrant activation of Notch1 helps cells acquire epithelial–mesenchymal transition and CSC self-renewal properties and is associated with pancreatic cancer treatment failure [24, 25]. However, the role and mechanism of Notch1 signaling in acquired gemcitabine resistance remain elusive. PI3K/AKT signaling is extensively activated in many tumors, including pancreatic cancers [26, 27]. AKT inhibition induces apoptosis of pancreatic cancer cells and enhances the killing effect of gemcitabine [28]. Moreover, there exists a reciprocal regulation between the Notch1 and AKT signaling pathways [29], by which both of them interactively regulate chemoresistance and maintenance of stemness.

In this study, we verified that the hypoxic niche synergistically enhances gemcitabine-induced stemness and acquired resistance in pancreatic cancer cells by activating the AKT/Notch1 signaling cascade. Furthermore, a chemotherapeutic combination involving the blockade of such a signaling pathway weakens the gemcitabine-enriched CSC population, providing a new therapeutic strategy against acquired chemoresistance.

## Methods

### Cell culture and treatments

The human pancreatic cancer cell line PANC-1 was obtained from the American Type Culture Collection (Manassas, VA, USA). The Patu8988 cell line was purchased from Nanjing KeyGen Biotech. Co. Ltd. (Nanjing, China). Both cell lines were cultured in Roswell Park Memorial Institute 1640 medium supplemented with 10% fetal bovine serum (FBS), 100 U/mL penicillin, and 100 μg/mL streptomycin, in a humidified incubator with 5% CO_2_ at 37 °C. After reaching a 60–80% confluence level, the cells were treated with different concentrations of gemcitabine (Selleck, Houston, TX, USA) for 24 h. To examine the role of the Notch1 or AKT signaling pathway in enhancing stemness, the pancreatic cancer cells were pretreated with 10 μM DAPT (γ-secretase inhibitor; Selleck) for 24 h or 20 μM LY294002 (AKT inhibitor; Beyotime Biotechnology, Shanghai, China) for 2 h before gemcitabine treatment. To clarify the effect of hypoxia on pancreatic cancer cell stemness, the cells were treated with 1% O_2_ for different time intervals or with various doses of CoCl_2_ (Sigma-Aldrich, St. Louis, MO, USA) for 24 h. To test the synergistic effect of hypoxia and gemcitabine, the cells were co-treated with optimal doses of gemcitabine and CoCl_2_ (as indicated in the pertinent figure legends) for 24 h.

### Western blot analysis

Western blot analysis was performed as previously described [13]. In brief, total cell lysates were electrophoresed in a sodium dodecyl sulfate–polyacrylamide gel electrophoresis gel and transferred onto polyvinylidene difluoride membranes (Millipore, Burlington, MA, USA). The membranes were blocked with 5% skim milk and incubated overnight with primary antibodies. After washing, the membranes were incubated with secondary antibodies conjugated with horseradish peroxidase, and the proteins were visualized by adding an enhanced chemiluminescence substrate (Thermo Fisher Scientific, Waltham, MA, USA). Antibodies against Bmi1, Notch1, NICD1, AKT, p-AKT (phosphorylated AKT), and GAPDH (glyceraldehyde 3-phosphate dehydrogenase) were purchased from Cell Signaling Technology (Danvers, MA, USA), and those against Sox2 and HIF-1α (hypoxia-inducible factor-1α) were purchased from Abcam (Boston, MA, USA).

### Transwell migration/invasion assay

Migration and invasion assays were performed in 24-well Transwell chambers (Corning, Fisher Scientific). For the transwell invasion assay, the upper compartment of the chamber was precoated with Matrigel (Sigma-Aldrich). Equal amounts of approximately 10 × 10^4^ cells were seeded into each upper chamber. The upper and lower chambers were filled with culture medium containing 0.1 and 30% FBS, respectively. After about 24 h, the migratory and invasive cells on the lower surface of the membrane were fixed, stained with 0.1% crystal violet, and then counted in five random fields under a light microscope.

### MTT assay

The MTT assay was performed as previously described [30]. After different treatments, the pancreatic cancer cells were seeded into 96-well plates and further incubated with various concentrations of gemcitabine (Selleck) for 48 h. Then, 20 μL of MTT solution (5 mg/mL; Sigma-Aldrich) was added to each well. The plates were incubated for 4 h, after which the medium was replaced with 150 μL of dimethyl sulfoxide (Sigma-Aldrich). The optical density was detected at 490 nm. Each concentration of gemcitabine was set up in five replicate wells.

### Flow cytometry analysis

Flow cytometry analysis was performed as previously described [13]. Anti-CD24–FITC antibody was purchased from BD Pharmingen (San Diego, CA, USA).

### Sphere-forming ability assay

The sphere-forming ability assay was performed in stem cell medium (SCM) as previously described [13]. Briefly, after different treatments, the pancreatic cancer cells were washed three times and suspended in SCM, which consisted of Dulbecco’s modified Eagle’s medium/F12 medium supplemented with bovine serum albumin (0.4%; Sigma-Aldrich), Insulin-Transferrin-Selenium (ITS; 1×; Sigma-Aldrich), basic fibroblast growth factor (10 ng/mL; PeproTech, Rocky Hill, NJ, USA), and epidermal growth factor (20 ng/mL; PeproTech). Approximately 1 × 10^4^ cells per well were seeded into ultralow-attachment 6-well plates (Corning), and the medium was changed every 3 days. After 15 to 20 days, the formed spheres (diameter ≥ 50 μm) were counted under a light microscope. The efficiency of sphere formation was calculated on the basis of the ratio of number of spheres to total number of cells.

### Tumor xenografts

Xenografts were formed by subcutaneously injecting PANC-1 cancer cells into the right flank of 3- to 4-week-old athymic mice (2 × 10^6^ cells per 100 μL per mouse) (HFK Bioscience Co., Beijing, China). Approximately 6 days after subcutaneous implantation, the mice were randomly separated into the control, GEM (gemcitabine), GEM+DAPT, and DAPT groups (*n* = 5 per group). Gemcitabine (20 mg/kg) and DAPT (10 mg/kg) were intraperitoneally injected every 3 days and every day, respectively. Tumor volume was measured periodically by using the following formula: Volume = 0.5 × length × width^2^. The experimental protocol complied with the “Guide for the Care and Use of Animals in Wuhan University”.

### In vivo lung metastasis assay

PANC-1 cells were separated into four groups (control, GEM, GEM+DAPT, and GEM+LY294002) and treated as indicated above. After treatment, approximately 4 × 10^6^ cells suspended in 0.2 mL phosphate-buffered saline were injected into the lateral tail vein of 7- to 8-week-old nude mice (HFK Bioscience Co.; *n* = 5 per group). After about 4 weeks, the mice were euthanized, and the lungs were completely resected and photographed. For hematoxylin and eosin (H&E) staining, the lungs were fixed with 4% paraformaldehyde and cut into 5-μm sections. The specimens were then stained with H&E, and the number of metastases was detected microscopically. All mice were handled in accordance with the protocols approved by the “Guide for the Care and Use of Animals in Wuhan University”.

### Statistical analysis

The data in our study were expressed as mean ± standard deviation. Student’s *t*-test was used to compare differences between two groups. Values were considered statistically significant at *P* < 0.05.

## Results

### Gemcitabine promotes Notch1 activation and pancreatic cancer cell stemness

**Fig. 1:**
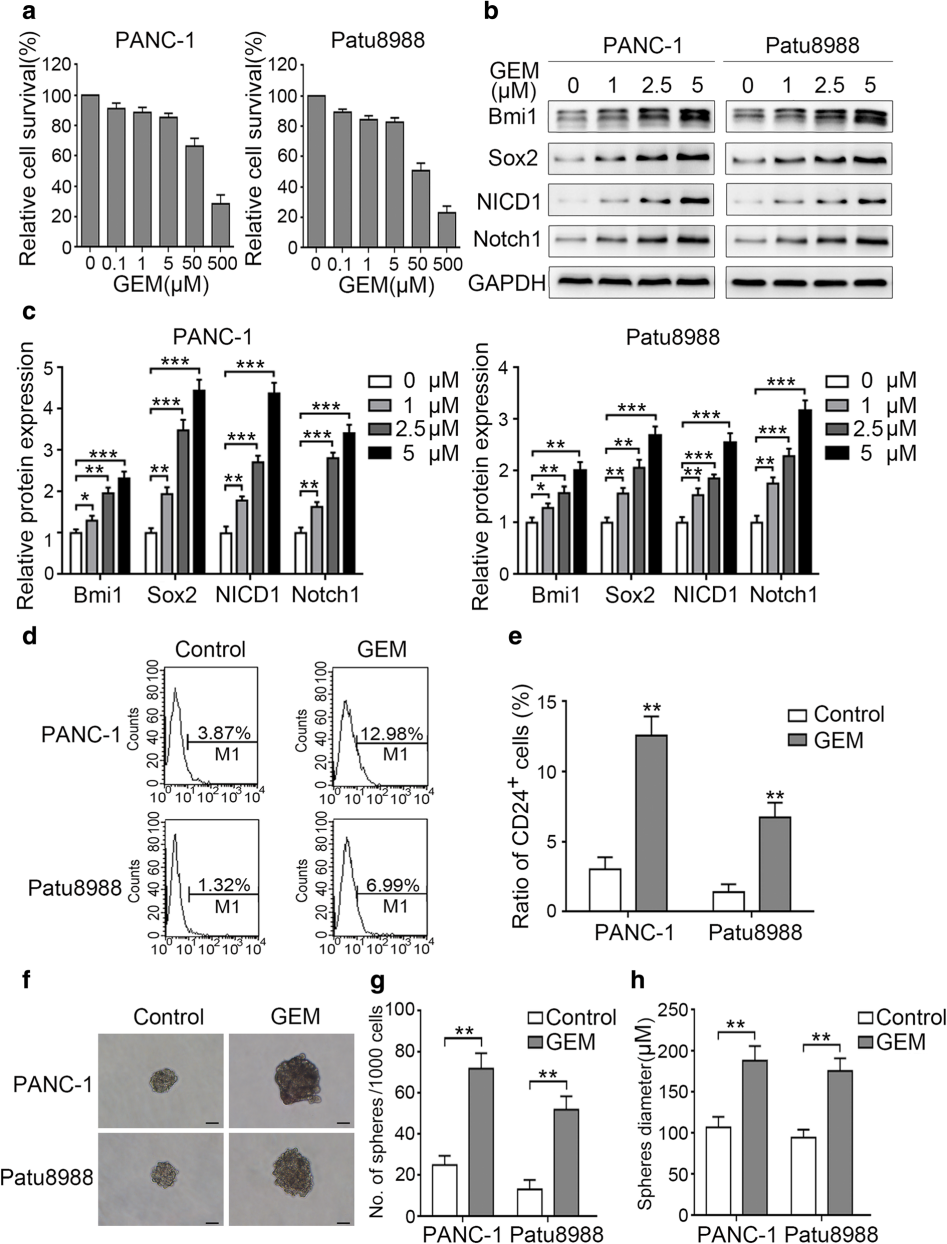
Gemcitabine promotes Notch1 activation and pancreatic cancer cell stemness**.** (**a**) PANC-1 and Patu8988 cells were treated with 0.1–500 μM gemcitabine for 24 h, and the relative survival rate was measured by the MTT assay. Western blot findings revealed (**b**) the representative expression levels of Bmi1, Sox2, NICD1, and Notch1 as well as (**c**) the changes in these levels after treatment with different concentrations of gemcitabine for 24 h. After treatment with 5 μM gemcitabine for 24 h, (**d**) the representative expression level of the pancreatic CSC marker CD24 as well as (**e**) the change in the proportion of CD24^+^ pancreatic CSCs were determined by FCM. (**f**-**h**) The ability of the cells to form spheres after treatment was evaluated by the sphere-forming assay in stem cell medium: (**f**) Representative image of sphere formation in cancer cells; (**g**, **h**) Charts showing the data on sphere number and diameter. The data are derived from three independent assays. Scale bar, 50 μm. *P < 0.05; **P < 0.01; ***P < 0.001

In our previous study, we had shown that low-dose gemcitabine treatment can enhance the stemness of pancreatic cancer cell lines SW1990 and BxPC-3 [13]. In the present study, we further analyzed whether gemcitabine has a similar effect on other pancreatic cancer cell lines such as PANC-1 and Patu8988. Our results revealed that low-dose gemcitabine treatment (1–5 μM) for 24 h, which has a minimal killing effect on pancreatic cancer cells (Fig. 1a), induced the expression of stemness-associated molecules Bmi1 and Sox2 as well as the CSC marker CD24 (Fig. 1b-e). In line with these changes, gemcitabine treatment also enhanced the sphere-forming ability of the evaluated cell lines, which exhibited a greater number of cell spheres and larger microsphere size after treatment (Fig. 1f-h). Although Notch1 signaling has been reported to play an important role in maintaining the stemness and self-renewal ability of CSCs [31], studies on the correlation between gemcitabine and Notch1 signaling are still lacking. Our results revealed that low-dose gemcitabine treatment promoted the expression of both Notch1 and NICD1 in a dose-dependent manner (Fig. 1b and c). Together, our results suggest that low-dose gemcitabine treatment activates Notch1 signaling and induces stemness in pancreatic cancer cells.

### Notch1 signaling mediates gemcitabine-induced stemness

**Fig. 2:**
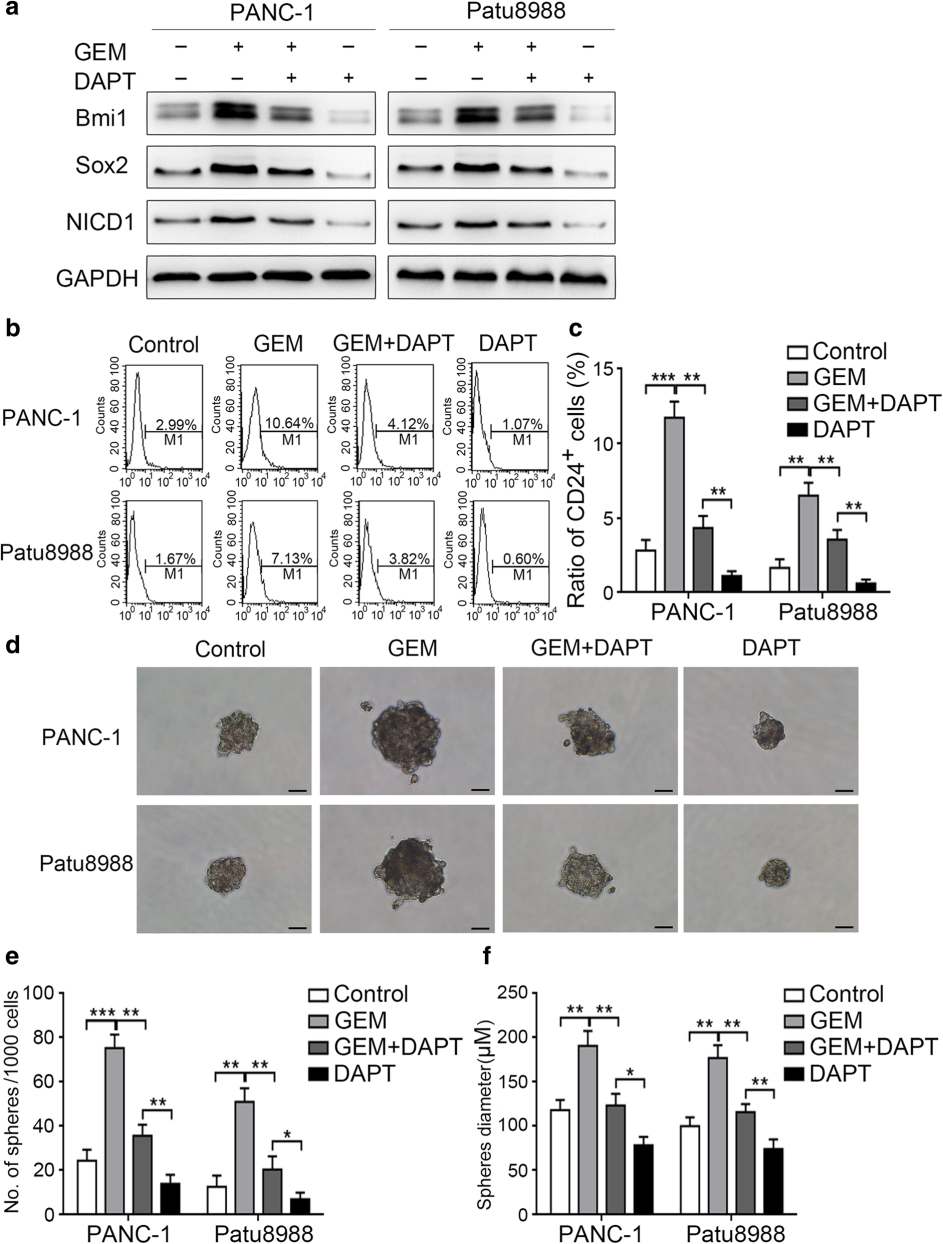
Notch1 signaling mediates gemcitabine-induced stemness. PANC-1 and Patu8988 cells were pretreated with 10 μM DAPT for 24 h and then treated with gemcitabine. (**a**) The expression levels of Bmi1, Sox2, and NICD1 were determined by Western blot analysis. (**b**) The representative expression level of the pancreatic CSC marker CD24 as well as (**c**) the change in the proportion of CD24^+^ pancreatic CSCs were determined by FCM. (**d**-**f**) The ability of the cells for sphere formation after treatment was determined by the sphere-forming assay: (**d**) Representative image of spheres formed after treatment; (**e**, **f**) Charts showing the data on sphere number and size. The results presented are from three independent assays. Scale bar, 50 μm. *P < 0.05; **P < 0.01; ***P < 0.001

To further confirm the role of Notch1 in gemcitabine-enhanced stemness, we pretreated pancreatic cancer cells with 10 μM γ-secretase inhibitor DAPT for 24 h before gemcitabine treatment. The Western blot findings showed that pretreatment with DAPT abolished gemcitabine-induced NICD1 expression (Fig. 2a). Further, Notch1 inhibition dramatically impaired the upregulation of Bmi1, Sox2, and CD24 expression (Fig. 2a-c). In addition, we observed a relative decrease in the number and size of spheres after Notch1 inhibition (Fig. 2d-f). It has been established that the properties of CSCs are connected with enhanced migration and invasion [32]. In the present study, we detected such changes after Notch1 inhibition. Our results showed that gemcitabine treatment increased the migratory and invasive abilities of pancreatic cancer cells, whereas suppression of Notch1 significantly abolished these increases (Additional file 1: Figure S1a-d). Moreover, pretreatment with DAPT dramatically reversed the gemcitabine-induced chemoresistance (Additional file 1: Figure S1e). These results show that gemcitabine promotes pancreatic cancer cell stemness and associated migration, invasion, and chemoresistance partly through Notch1 activation.

### Notch1 inhibition enhances the killing effect of gemcitabine and suppresses metastasis in vivo

**Fig. 3:**
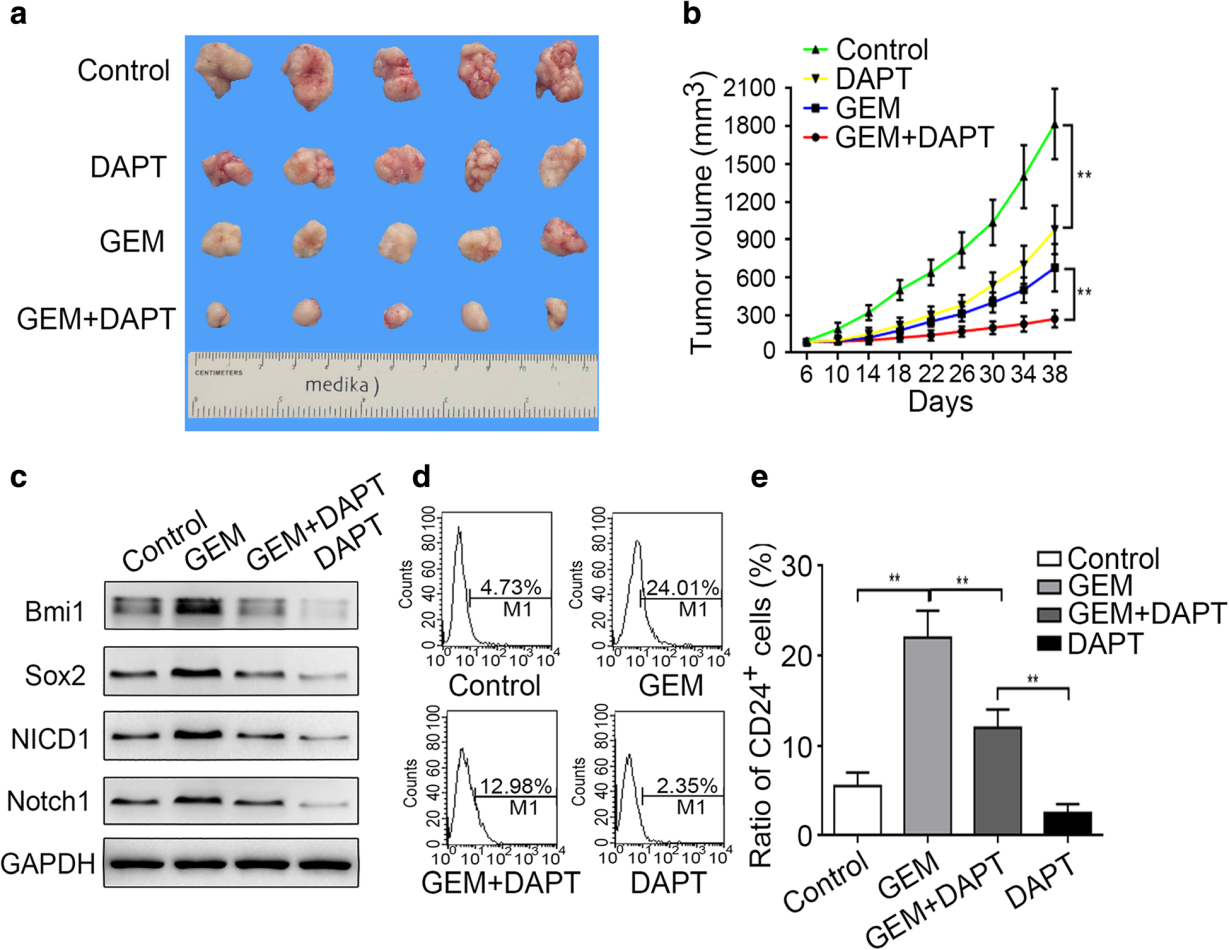
Notch1 inhibition enhances the killing effect of gemcitabine in vivo. PANC-1 cells were subcutaneously injected into the right flank of nude mice. After about 6 days, the mice were randomly divided into the control, DAPT, GEM, and GEM+DAPT groups in accordance with the protocol described in the Methods. (**a**) Representative tumor size at 38 days post-treatment. (**b**) Tumor growth curves delineated on the basis of volume measured every 4 days. (**c**) After treatment, the expression levels of Bmi1, Sox2, NICD1, and Notch1 were determined by Western blot analysis. (**d**, **e**) Tumor samples were digested by using collagenase I, and the change in the proportion of CD24^+^ pancreatic CSCs was determined by FCM. The graphs are from three independent experiments. **P < 0.01

Because Notch1 activation was revealed to play a role in gemcitabine-induced stemness and associated malignant traits, we next investigated the effect of supplementation with Notch1 inhibition on chemosensitivity in vivo. As shown in Fig. 3a and b, DAPT treatment significantly reduced the tumor growth rate and size relative to the control at 38 days post-treatment. When combined with gemcitabine chemotherapy, DAPT treatment also synergistically strengthened the killing effect of gemcitabine in pancreatic cancer cells. We further examined the changes in Bmi1 and Sox2 expression and CD24^+^ cell population at the end of treatment. As shown in Fig. 3c-e, gemcitabine chemotherapy increased the expression levels of Bmi1 and Sox2 as well as the proportion of CD24^+^ cells, while combination treatment with DAPT abolished these enrichments. CSCs have an inherent potential for metastasis [33, 34]. Our results, too, revealed an enhanced ability of the cells for lung metastasis after gemcitabine treatment, which was attenuated when combined with DAPT treatment (Additional file 2: Figure S2a-c). These results show that Notch1 inhibition synergistically potentiates the killing effect of gemcitabine and suppresses metastasis in vivo.

### AKT promotes pancreatic cancer cell stemness partly by mediating Notch1 activation

**Fig. 4:**
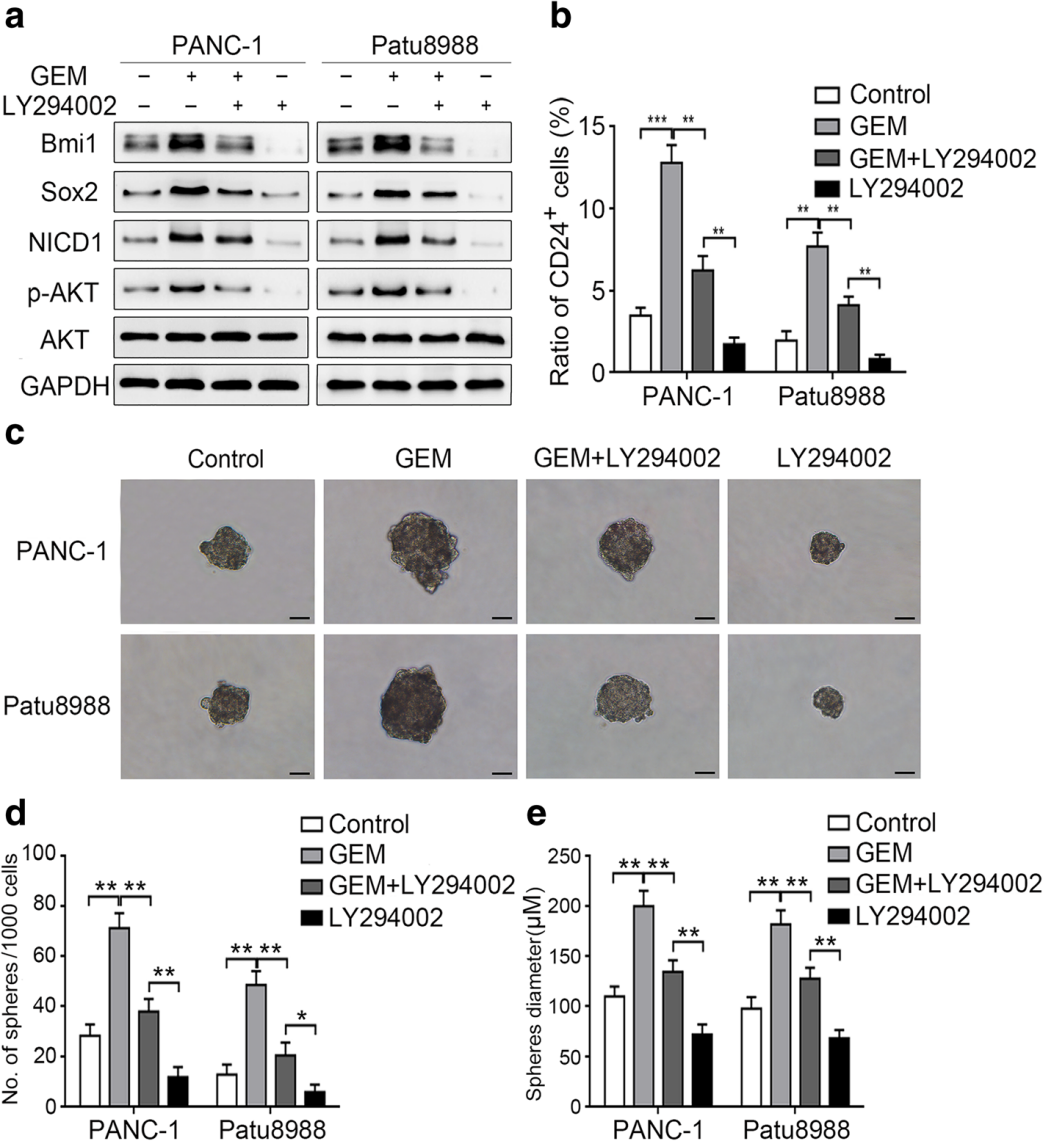
AKT promotes pancreatic cancer cell stemness partly by mediating Notch1 activation. Two cell lines were pretreated with 20 μM LY294002 for 2 h and then treated with gemcitabine. (**a**) The expression levels of Bmi1, Sox2, NICD1, p-AKT (serine 473), and AKT were determined by Western blot analysis. (**b**) The change in the proportion of CD24^+^ pancreatic CSCs was determined by FCM. (**c**-**e**) The ability of the cells for sphere formation was investigated by the sphere-forming assay: (**c**) Representative image of spheres formed after treatment; (**d** and **e**) Charts showing the data on sphere number and size. The graphs show the results of three independent experiments. Scale bar, 50 μm. *P < 0.05; **P < 0.01; ***P < 0.001

AKT is commonly activated in pancreatic cancer and participates in gemcitabine chemoresistance, and inhibition of AKT could enhance the killing effect of gemcitabine [35]. Our results revealed that gemcitabine treatment promoted the expression of p-AKT (serine 473) in PANC-1 and Patu8988 cell lines (Fig. 4a). To determine the role of AKT in gemcitabine-induced stemness, we pretreated the pancreatic cancer cells with 20 μM LY294002 (an AKT inhibitor) for 2 h before gemcitabine treatment. As indicated in Fig. 4a, AKT inhibition significantly suppressed gemcitabine-induced AKT activation. Subsequently, the expression of Bmi1, Sox2, and CD24 was significantly impaired (Fig. 4a and b). Further, LY294002 pretreatment attenuated the gemcitabine-induced sphere-forming ability of the pancreatic cancer cells (Fig. 4c-e). We further examined the role of AKT in Notch1 activation after gemcitabine treatment. Our results demonstrated that LY294002 attenuated gemcitabine-induced NICD1 expression in both cancer cell lines (Fig. 4a). Then, we analyzed the changes in the stemness-related metastatic, migratory, and invasive abilities of cancer cells after AKT inhibition. Our results showed that pretreatment with LY294002 markedly attenuated gemcitabine-enhanced metastasis in vivo (Additional file 2: Figure S2a-c). It also weakened the migratory and invasive abilities of pancreatic cancer cells (Additional file 3: Figure S3a-d). In total, our results suggest that AKT plays a role in promoting gemcitabine-induced Notch1 activation and stemness.

### Hypoxia synergistically enhances gemcitabine-induced stemness

**Fig. 5:**
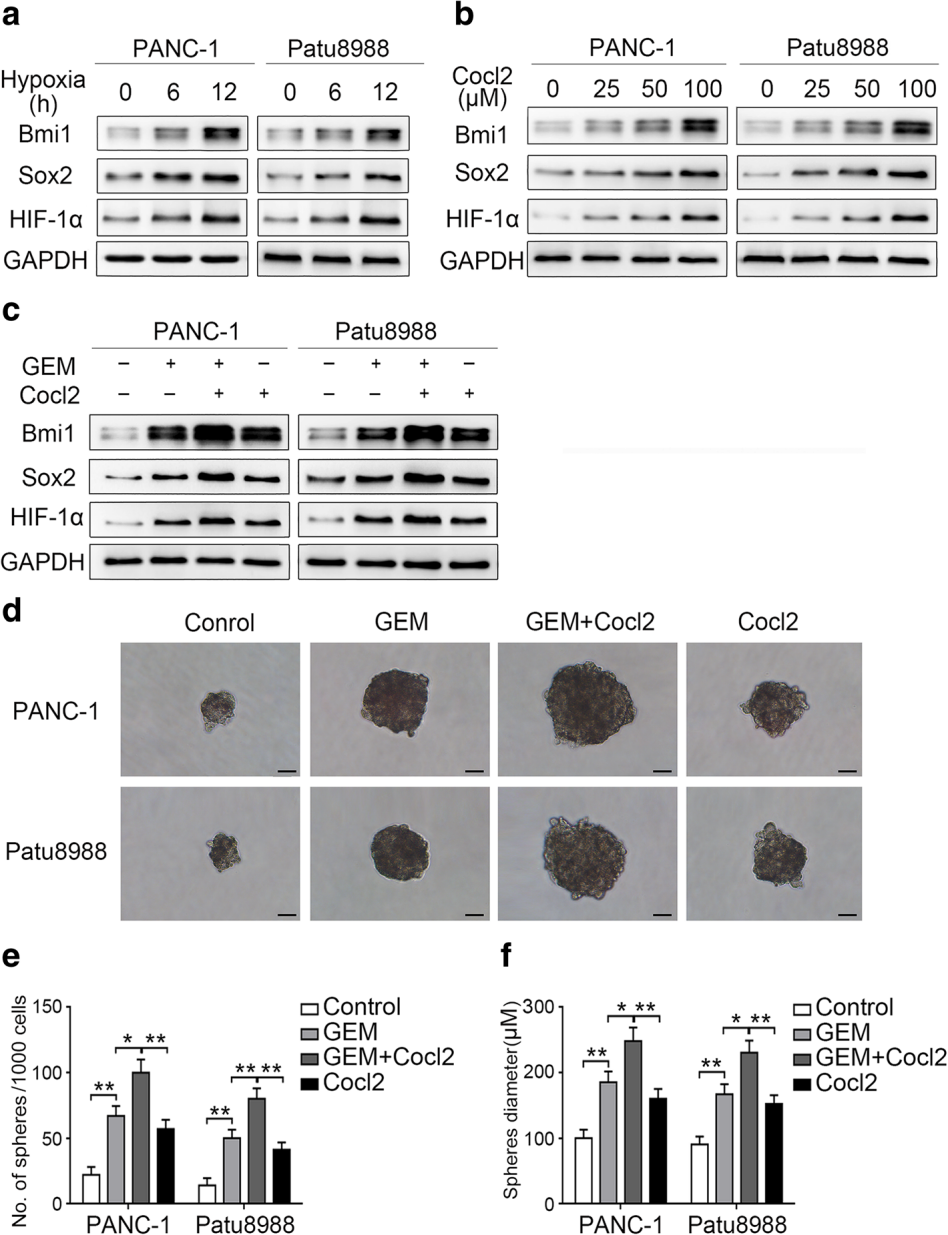
Hypoxia synergistically enhances gemcitabine-induced stemness. (**a**) Two pancreatic cancer cell lines were incubated under different hypoxic (1%) conditions, and the expression levels of Bmi1, Sox2, and HIF-1α were determined by Western blot analysis. (**b**) After treatment with different concentrations of CoCl_2_ for 24 h, the changes in Bmi1, Sox2, and HIF-1α expression levels were determined by Western blot analysis. (**c**) Two cell lines were co-treated with gemcitabine and CoCl_2_ for 24 h, and the protein expression levels were measured by Western blot analysis. (**d**) The sphere formation ability of the cells after co-treatment was determined by the sphere-forming assay. (**e**, **f**) Charts showing the data on sphere number and size after treatment. The graphs are from three independent experiments. Scale bar, 50 μm. *P < 0.05; **P < 0.01

It is well known that hypoxia is a prominent feature of the microenvironment in pancreatic cancer and that it enhances the chemoresistance against gemcitabine [14]. We, therefore, analyzed the effect of hypoxia on stemness. As shown in Fig. 5a, treatment of pancreatic cancer cells under hypoxic (1%) conditions for 6–12 h significantly promoted the expression of HIF-1α, an important marker in hypoxia response. In addition, stemness-associated molecules Bmi1 and Sox2 were upregulated after hypoxia treatment. We also treated pancreatic cancer cells with CoCl_2_, a chemical which stabilizes HIF-1α, to mimic a hypoxic microenvironment. CoCl_2_treatment for 24 h, too, promoted Bmi1 and Sox2 expression in a dose-dependent manner in both evaluated cells (Fig. 5b). To further verify the synergistic effect of hypoxia and gemcitabine treatment on stemness induction, we co-treated pancreatic cancer cells with gemcitabine and CoCl_2_ for 24 h. The Western blot findings showed that combination treatment with CoCl_2_ further reinforced the gemcitabine-inductive effect on Bmi1 and Sox2 (Fig. 5c). In accord, CoCl_2_ treatment also enhanced the number and size of gemcitabine-induced spheres (Fig. 5d-f). Our results suggest that hypoxia synergistically enhances gemcitabine-induced stemness.

### AKT/Notch1 signaling mediates the synergistic enhancement of the stemness induced by gemcitabine and hypoxia co-treatment

**Fig. 6:**
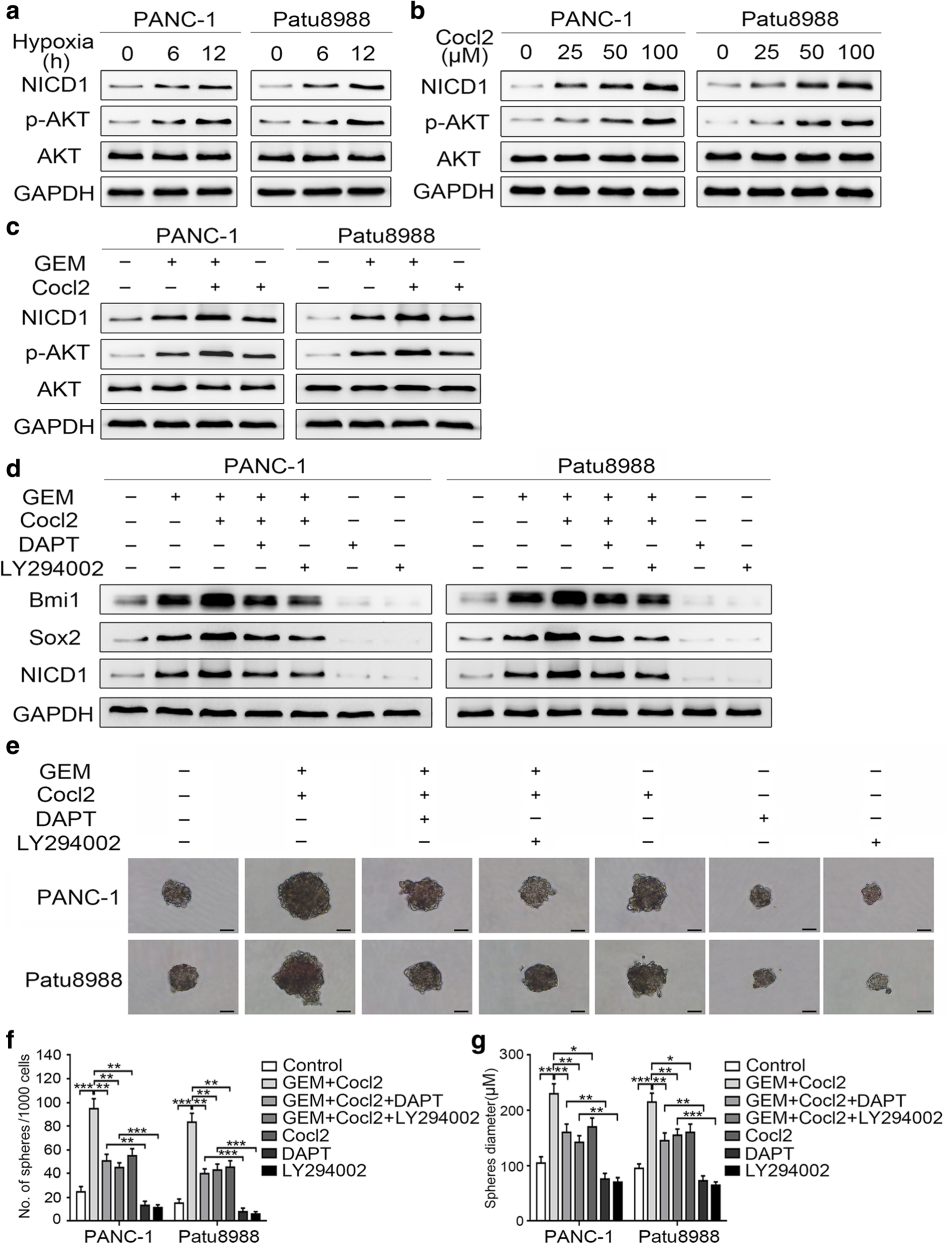
AKT/Notch1 signaling mediates the synergistic enhancement of stemness induced by gemcitabine and hypoxia. (**a**) PANC-1 and Patu8988 cells were treated under hypoxic conditions (1%) for different durations, and the expression levels of NICD1, p-AKT, and AKT were determined by Western blot analysis. (**b**) After treatment with different concentrations of CoCl_2_, the changes in NICD1, p-AKT, and AKT expression levels were determined by Western blot analysis. (**c**) Two cell lines were co-treated with gemcitabine and CoCl_2_, and the protein expression levels were determined by Western blot analysis. (**d**) Two pancreatic cancer cell lines were pretreated with 10 μM DAPT or 20 μM LY294002 and then treated with a combination of gemcitabine and CoCl_2_. Then, the expression levels of Bmi1, Sox2, and NICD1 were determined by Western blot analysis. (**e**-**g**) After this treatment, the two cell lines were cultured with stem cell medium, and the ability of the cells for sphere formation was investigated by the sphere-forming assay: (**e**) Representative image of spheres after treatment; (**f, g**) Charts showing the data on sphere number and size. All data shown are from three independent experiments. Scale bar, 50 μm. *P < 0.05; **P < 0.01; ***P < 0.001

Because Notch1 has been demonstrated to mediate gemcitabine-induced stemness, we next analyzed the changes on the basis of hypoxic status. The Western blot findings revealed that both hypoxia and CoCl_2_ treatment increased the expression of NICD1 (Fig. 6a and b). The synergistically inductive effect of these two treatments was more obvious when combined with gemcitabine treatment (Fig. 6c). We also pretreated pancreatic cancer cells with DAPT before co-treatment with gemcitabine and CoCl_2_. The results showed that DAPT significantly attenuated the synergistic enhancement of Bmi1 and Sox2 expression (Fig. 6d). In addition, Notch1 inhibition significantly suppressed the synergistic enhancement of sphere number and size (Fig. 6e-g). These results suggested that Notch1 activation plays a role in the synergistic enhancement of stemness induced by combination treatment with gemcitabine and CoCl_2_.

Hypoxia has been reported to activate AKT expression [14]. Our results also demonstrated a similar effect in pancreatic cancer cells (Fig. 6a and b). In addition, synergistically augmented p-AKT expression was more evident after gemcitabine and CoCl_2_ co-treatment (Fig. 6c), whereas AKT suppression significantly suppressed the collaboratively inductive effect of the co-treatment on Bmi1 and Sox2 expression (Fig. 6d). In line with these changes, the enhanced sphere-forming ability of the cells was also dramatically reduced after AKT inhibition (Fig. 6e-g). Moreover, the induction of NICD1 expression by gemcitabine and CoCl_2_ co-treatment was significantly abolished by pretreatment with an AKT inhibitor in both evaluated cells (Fig. 6d), which suggests the role of AKT regulation in Notch1 activation in cells treated with gemcitabine and CoCl_2_. Collectively, these results suggest that AKT/Notch1 signaling plays a role in promoting the synergistic induction of stemness by gemcitabine and hypoxia.

## Discussion

**Fig. 7:**
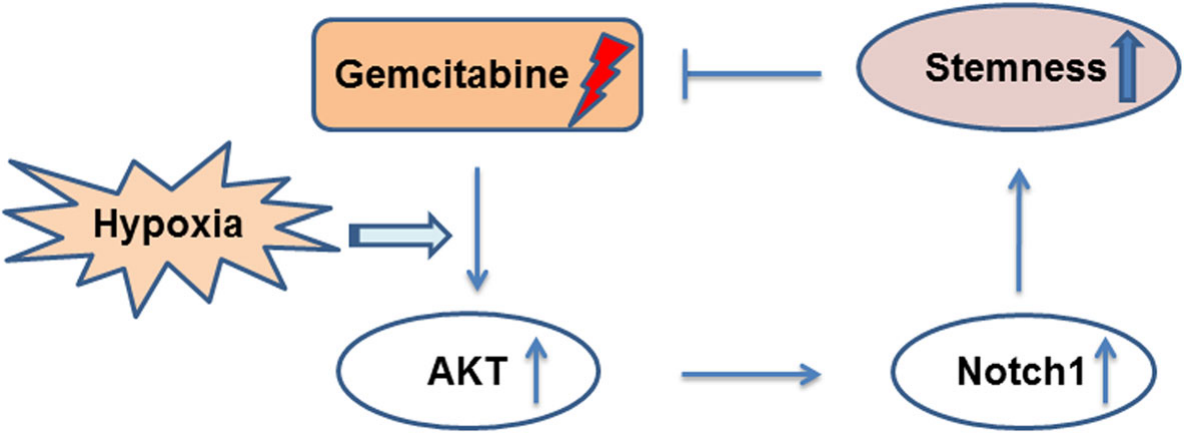
Schematic illustration demonstrating how hypoxia potentiates gemcitabine-induced stemness and acquired resistance in pancreatic cancer cells through AKT/Notch1 signaling

Resistance to chemotherapy is an intractable problem in pancreatic cancer treatment. Gemcitabine is a standard first-line chemotherapeutic agent. However, its clinical benefit has remained dismal during the past decades. It has been suggested that a hypoxic microenvironment enhances the resistance of cells to gemcitabine chemotherapy. CSCs, or tumor-initiating cells, are a small subset of cancer cells that display high tumorigenicity and great resistance to drugs [36, 37]. In this study, we showed that low-dose gemcitabine treatment promotes the stemness of pancreatic cancer cells, and this effect is synergistically enhanced by the hypoxic niche. Moreover, our results suggested that the AKT/Notch1 signaling cascade partly mediates this process (Fig. 7). Additionally, our in vivo findings demonstrated that pharmaceutical inhibition of Notch1 signaling enhances the killing effect of gemcitabine in pancreatic cancer cells. Together, our data put forth a new potential mechanism of gemcitabine resistance and offer potential targets to enhance the chemosensitivity of cells to gemcitabine.

The Notch1 signaling pathway plays an essential role in the maintenance and self-renewal of CSCs in a variety of malignancies [38, 39]. It has been suggested that a fraction of pancreatic CSCs are enriched as a consequence of the killing effect of gemcitabine treatment, with a small residual cells exhibiting an activated Notch1 signaling pathway [25]. In our study, gemcitabine (a relatively low dose) might not have had a cytotoxic effect because of the high rate of cell survival after gemcitabine treatment. We found that gemcitabine treatment increased Notch1 and NICD1 expression in a dose-dependent manner. Therefore, we believe that the activation of Notch1 resulted mainly from the “inductive effect” of gemcitabine and not just from the “enrichment” of CSCs. In addition, pretreatment with the γ-secretase inhibitor DAPT significantly inhibited the molecules and behaviors (such as migration, invasion, and chemoresistance) associated with gemcitabine-induced stemness. Further, combination treatment with DAPT enhanced the killing effect of gemcitabine and inhibited gemcitabine-induced metastasis in vivo. Our results suggest that induction of Notch1 signaling plays an important role in gemcitabine-enhanced stemness.

AKT activation plays an important role in the migration, invasion, metastasis, chemoresistance, and CSC-like phenotype of pancreatic cancer cells, and it is closely correlated with the prognosis [27, 40–42]. AKT activation overlaps in function and entails mutual cooperation with Notch1 [29, 43]. In our study, we showed that low-dose gemcitabine can facilitate AKT activation, which is in line with our findings on the enhanced stemness of pancreatic cancer cells. Further, AKT inhibition significantly attenuated gemcitabine-activated Notch1 expression as well as the consequent CSC-like behaviors of the cells. Our results suggest a potentially adverse collaboration between Notch1 and AKT, in which the two signaling pathways are intertwined in increasing the stemness of pancreatic cancer cells upon gemcitabine chemotherapy. Therefore, our results are consistent with previous findings and suggest the role of AKT signaling in promoting gemcitabine-induced Notch1 activation and pancreatic cancer cell stemness.

Hypoxia is a frequent condition in pancreatic cancer. It contributes greatly to the apoptosis resistance of cells to chemotherapy [14]. However, the exact mechanism remains obscure. Increasing evidence has demonstrated that the hypoxic niche plays an important role in maintaining the self-renewal property and undifferentiated condition of various CSCs [18, 44]. In our study, we also showed that hypoxia synergistically promotes the gemcitabine-induced CSC-like phenotype, with an increased expression of stemness-associated molecules and enhancement of sphere-forming ability, which might explain the persistence of gemcitabine resistance in pancreatic cancer. Hypoxia activates Notch-responsive promoters and promotes the expression of downstream genes [45]. Our study also demonstrated an increase in both NICD1 expression and AKT activation after hypoxia treatment. In addition, inhibition of AKT abolished Notch1 activation and pancreatic cell stemness induced by co-treatment with gemcitabine and hypoxia. Therefore, our results demonstrate that hypoxia collaboratively promotes gemcitabine-induced stemness through the AKT/Notch1 signaling pathway.

## Conclusions

In conclusion, our data show a novel mechanism of acquired gemcitabine resistance in pancreatic cancer through stemness induction, which is aggravated by the ubiquitous hypoxic niche in cancer cells. Thus, strategies aimed at eliminating pancreatic CSCs might present a promising approach for overcoming gemcitabine resistance and developing effective treatments for pancreatic cancer. Moreover, our results highlight the important role of the AKT/Notch1 signaling pathway in mediating this process. We provide evidence that combination treatment with adjuvant drugs targeting such signaling pathways offers a better therapeutic benefit against pancreatic cancer in vitro and in vivo, suggesting AKT/Notch1 as attractive targets for eliminating pancreatic CSCs. Altogether, our study provides new insights into strategies for reversing chemoresistance in patients with pancreatic cancer.

## Additional files

Additional file 1: **Figure S1.** Notch1 inhibition abolishes gemcitabine-enhanced migration, invasion, and chemoresistance. PANC-1 and Patu8988 cells were pretreated with 10 μM DAPT for 24 h and then treated with gemcitabine. (**a**, **b**) The transwell migration assay was performed to examine the change in the migratory ability of the cells, and the relative migratory ability was calculated by counting the number of stained cells migrating to the lower chamber. (**c**, **d**) The invasive ability of the cells was measured by the transwell invasion assay. (**e**) After treatment, the MTT assay was performed to test the change in the chemosensitivity of pancreatic cancer cells to gemcitabine. The graphs show the results of three independent experiments. Scale bar, 100 μm. *P < 0.05; **P < 0.01. (TIF 2082 kb)

Additional file 2: **Figure S2.** AKT/Notch1 inhibition abolishes gemcitabine-induced metastasis. Non-treated (control) and treated (GEM, GEM+DAPT, and GEM+LY294002) PNAC-1 cells were injected into the tail vein of nude mice. (**a**) Representative examples of resected lungs in each group at 4 weeks post-treatment. (**b**) Representative images of H&E staining of resected lungs in each group. The arrows indicate metastatic nodules. (**c**) The mean number of lung metastases was determined. Scale bar, 200 μm. **P < 0.01; ***P < 0.001. (TIF 2383 kb)

Additional file 3: **Figure S3.** AKT suppression attenuates gemcitabine-enhanced migratory and invasive abilities. Two pancreatic cancer cell lines were pretreated with 20 μM LY294002 for 2 h and then treated with gemcitabine. (**a**, **b**) The migratory ability of the cells was evaluated by the transwell migration assay, and the relative migratory ability was calculated by determining the number of cells migrating to the lower chamber under microscopic observation. (**c**, **d**) The transwell invasion assay was performed to measure the change in relative invasive ability. The graphs shown are from three independent experiments. Scale bar, 100 μm. **P < 0.01. (TIF 2126 kb)

## Abbreviations

CSCs: Cancer stem cells
FBS: Fetal bovine serum
FCM: Flow cytometry
GAPDH: Glyceraldehyde 3-phosphate dehydrogenase
GEM: Gemcitabine
HIF-1α: Hypoxia-inducible factor-1α
NICD1: NOTCH1 intracellular domain
SCM: Stem cell medium
