# Supplementary figures and images for: Hypoxia potentiates gemcitabine-induced stemness in pancreatic cancer cells through AKT/Notch1 signaling
Source: J Exp Clin Cancer Res. 2018 Nov 28;37:291. doi: 10.1186/s13046-018-0972-3 (PMC6263055; doi:10.1186/s13046-018-0972-3)

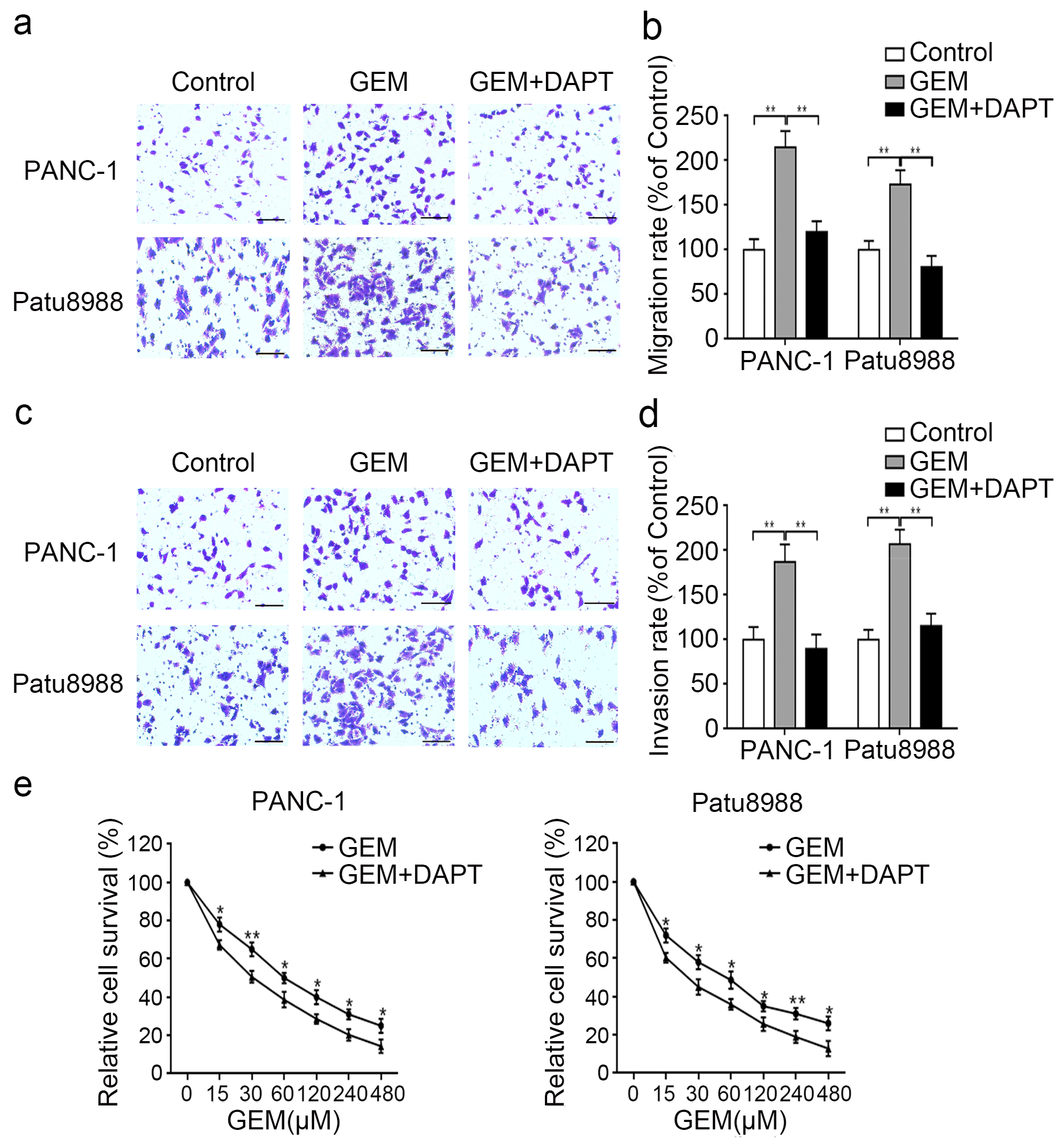

Supplement: Supplementary file 1 — Figure S1. Notch1 inhibition abolishes gemcitabine-enhanced migration, invasion, and chemoresistance. PANC-1 and Patu8988 cells were pretreated with 10 μM DAPT for 24 h and then treated with gemcitabine. (a, b) The transwell migration assay was performed to examine the change in the migratory ability of the cells, and the relative migratory ability was calculated by counting the number of stained cells migrating to the lower chamber. (c, d) The invasive ability of the cells was measured by the transwell invasion assay. (e) After treatment, the MTT assay was performed to test the change in the chemosensitivity of pancreatic cancer cells to gemcitabine. The graphs show the results of three independent experiments. Scale bar, 100 μm. *P < 0.05; **P < 0.01. (TIF 2082 kb) [file 13046_2018_972_MOESM1_ESM.tif]

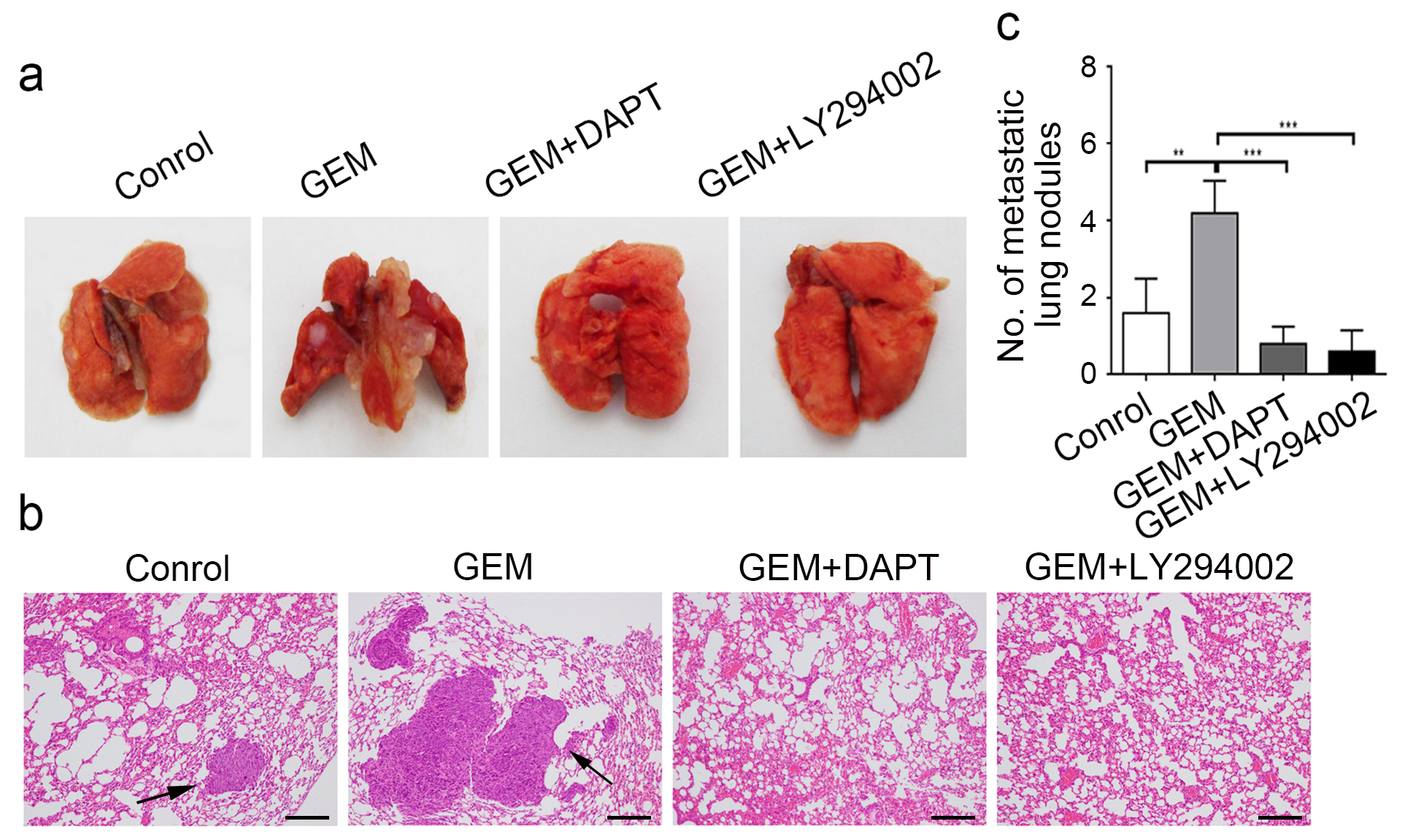

Supplement: Supplementary file 2 — Figure S2. AKT/Notch1 inhibition abolishes gemcitabine-induced metastasis. Non-treated (control) and treated (GEM, GEM+DAPT, and GEM+LY294002) PNAC-1 cells were injected into the tail vein of nude mice. (a) Representative examples of resected lungs in each group at 4 weeks post-treatment. (b) Representative images of H&E staining of resected lungs in each group. The arrows indicate metastatic nodules. (c) The mean number of lung metastases was determined. Scale bar, 200 μm. **P < 0.01; ***P < 0.001. (TIF 2383 kb) [file 13046_2018_972_MOESM2_ESM.tif]

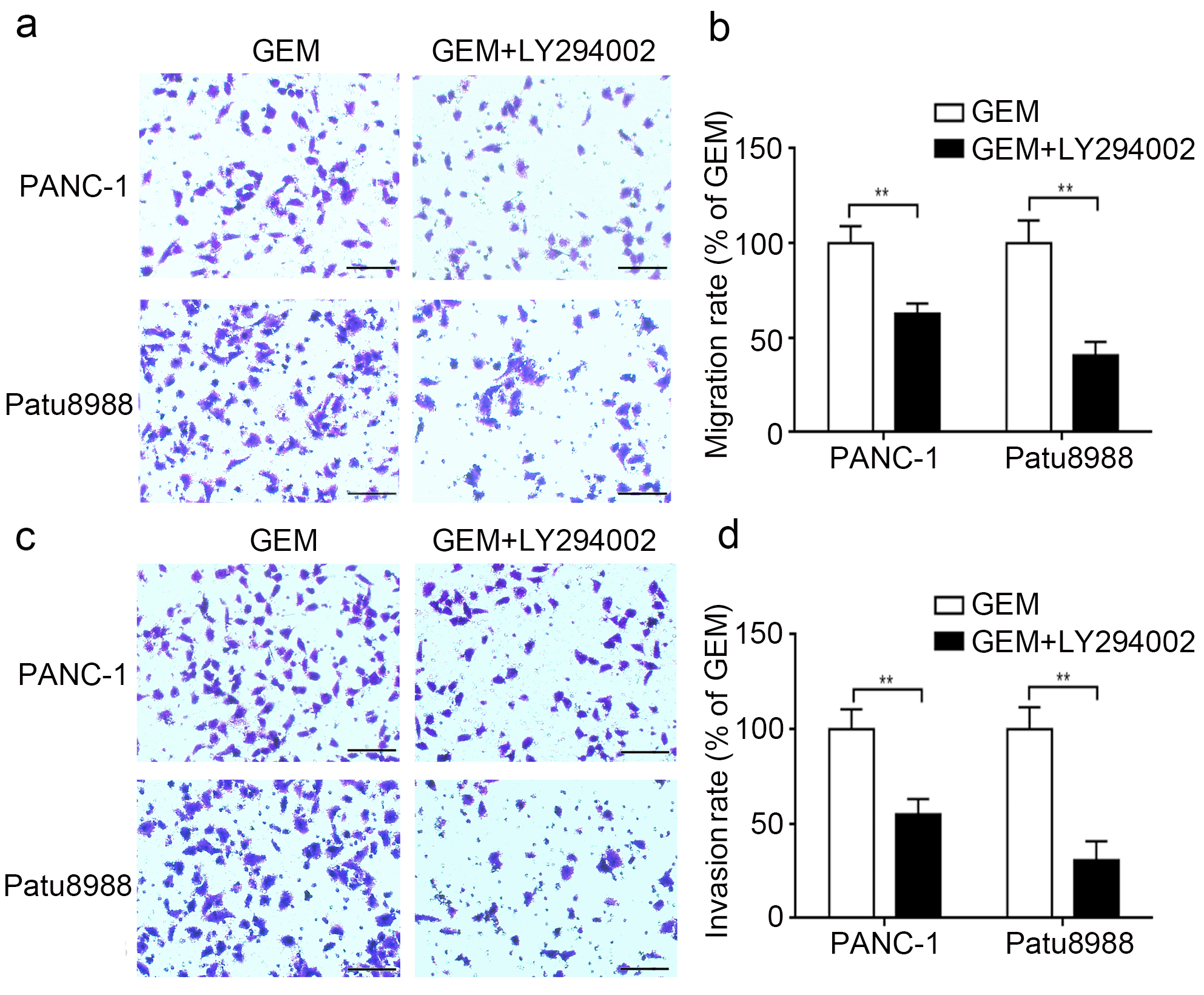

Supplement: Supplementary file 3 — Figure S3. AKT suppression attenuates gemcitabine-enhanced migratory and invasive abilities. Two pancreatic cancer cell lines were pretreated with 20 μM LY294002 for 2 h and then treated with gemcitabine. (a, b) The migratory ability of the cells was evaluated by the transwell migration assay, and the relative migratory ability was calculated by determining the number of cells migrating to the lower chamber under microscopic observation. (c, d) The transwell invasion assay was performed to measure the change in relative invasive ability. The graphs shown are from three independent experiments. Scale bar, 100 μm. **P < 0.01. (TIF 2126 kb) [file 13046_2018_972_MOESM3_ESM.tif]
